# Changes in Dietary Inflammatory Index Score over Time and Cancer Development in Rural Post-Menopausal Women

**DOI:** 10.3390/antiox12040946

**Published:** 2023-04-18

**Authors:** Mariah Kay Jackson, Joan Lappe, Jihyun Ma, Megan Timmerman, Elizabeth R. Lyden, Nitin Shivappa, James R. Hébert, Dianne Travers Gustafson, Laura Graeff-Armas, Corrine Hanson

**Affiliations:** 1 Medical Nutrition, Department of Medical Sciences, College of Allied Health Professions, University of Nebraska Medical Center, Omaha, NE 68198, USA; 2 College of Nursing, Creighton University, Omaha, NE 68178, USA; 3 Department of Biostatistics, College of Public Health, University of Nebraska Medical Center, Omaha, NE 68198, USA; 4 Department of Epidemiology and Biostatistics and Cancer Prevention and Control Program, University of South Carolina, Columbia, SC 29208, USA; 5 Division of Diabetes, Endocrine & Metabolism, Department of Internal Medicine, College of Medicine, University of Nebraska Medicine, Omaha, NE 68198, USA

**Keywords:** dietary assessment, DII, cancer, cancer survivorship, inflammation

## Abstract

Inflammation plays a key role in cancer development. As an important modulator of inflammation, the role of diet should be explored. The purpose of this study was to determine the association between diets with a higher inflammatory potential, as measured by the Dietary Inflammatory Index (DII^®^), and cancer development in a cohort of rural post-menopausal women. Dietary intake from a randomized controlled trial cohort of rural, post-menopausal women in Nebraska was used to compute energy-adjusted DII (E-DII^TM^) scores at baseline and four years later (visit 9). A linear mixed model analysis and multivariate logistic regression evaluated the association between E-DII scores (baseline, visit 9, change score) and cancer status. Of 1977 eligible participants, those who developed cancer (*n* = 91, 4.6%) had a significantly larger, pro-inflammatory change in E-DII scores (Non-cancer: Δ 0.19 ± 1.43 vs. Cancer: Δ 0.55 ± 1.43, *p* = 0.02). After adjustment, odds of cancer development were over 20% higher in those with a larger change (more pro-inflammatory) in E-DII scores than those with smaller E-DII changes (OR = 1.21, 95% CI [1.02, 1.42], *p* = 0.02). Shifting to a more pro-inflammatory diet pattern over four years was associated with increased odds of cancer development, but not with E-DII at baseline or visit 9 alone.

## 1. Introduction

Chronic inflammation is a central feature of cancer, known to promote genetic and epigenetic changes and tumor progression [1,2,3]. Overall diet patterns have shown robust associations with the development of cancer and have been investigated as a modifiable risk factor of inflammation. Mediterranean diet patterns have been extensively studied for their role in inflammation prevention and found to be linked to anti-inflammatory properties [4,5,6,7]. In contrast, Western-type diet patterns have been associated with increased pro-inflammatory markers, including C-reactive protein (CRP), interleukin-6 (IL-6), and fibrinogen [8,9,10]. The Dietary Inflammatory Index (DII^®^) was developed and validated to characterize and quantify cumulative dietary inflammatory potential [11]. As opposed to previous studies that have evaluated certain dietary patterns, the DII itself is not a dietary pattern. Rather, the DII takes into consideration the synergistic effect of multiple individual nutrients and the inflammatory potential of the diet. DII scores have been shown to positively correlate with markers of inflammation such as hs-CRP, as well as cancer outcomes across a variety of populations, including post-menopausal women in the US, Iran, Italy, and Sweden [12,13,14,15,16,17]. Post-menopausal women are generally at an age that increases their risk for cancer, as 80% of all cancers in the United States occur in people who are 55 years old or greater, making them an important target population [18].

Western-type diet patterns, marked by high consumption of red meat, high-fat dairy products, and refined grains, are widely generalized to describe the American diet. However, there is evidence to suggest that diets in the United States may differ based on rural versus urban inhabitance. Rural dietary patterns may be uniquely impacted by food access, education, and financial resources, leading to diets higher in sweets, starches, and high-fat foods compared to urban counterparts whose diets are higher in fruits and vegetables [19]. These discrepancies by geographic residence have been associated with higher prevalence of obesity in rural versus urban residents [20], a known contributor to inflammation and many cancers, such as post-menopausal breast cancer [3,13]. As rural-urban disparities exist, it is important to continue establishing key contributing factors, such as diet, to promote better outcomes in these at-risk populations [20,21].

Although diet plays a key role in modulating inflammation, the relationship between dietary inflammatory potential and cancer outcomes in certain populations, such as rural post-menopausal women, remains poorly understood. Therefore, the aim of this study was to analyze the diets of rural, post-menopausal women for inflammatory potential via DII/E-DII scores and their relationship to cancer development. The hypothesis is that more pro-inflammatory diets, evidenced by higher DII scores, are associated with the development of all-type cancers in rural post-menopausal women.

## 2. Materials and Methods

*Study population*: The present study uses an existing cohort of participants previously enrolled in a completed randomized controlled trial (RCT) evaluating the effect of a four-year vitamin D and calcium supplementation intervention on cancer development in rural, post-menopausal women in Nebraska from June 2009 to August 2015. Further details of the study design have been previously detailed [22]. In short, independently living post-menopausal women aged 55 years or older from 31 rural Nebraskan counties were screened for inclusion into the RCT. Women aged 55 and older were targeted as a population likely to be post-menopausal and an at-risk group for cancer due to age within the parent study. Post-menopausal was defined as at least four years past the last menses. Women were excluded for (1) any history of cancer diagnosis, unless it was a superficial basal or squamous cell carcinoma of the skin or a previous curatively treated malignancy that was resolved 10 or more years prior to the study; (2) history of chronic kidney disease; or (3) previous participation in a calcium/vitamin D study [22]. After meeting inclusion and exclusion criteria, participants were consented and randomized into either a placebo group or active treatment group of vitamin D (2000 IU) and calcium (1500 mg divided into 500 mg, three times daily). The primary outcome was any cancer assessed at 6-month intervals over four years. Within the RCT, a food frequency questionnaire (FFQ) was completed at the baseline visit and at the end of the four-year trial (visit 9). The RCT study was approved by the Institutional Review Board (IRB) of Creighton University (#624917-9-03; 8/5/2008), obtained informed consent from all participants, and is registered on clinicaltrials.gov (NCT01052051). Utilizing this trial, we conducted a secondary analysis, examining the association between dietary inflammatory potential over time and cancer development. In the present study, participants were included if they completed both FFQs in order to compute baseline and visit 9 DII scores.

*Dietary Inflammatory Index*: The Block Food Frequency Questionnaire (FFQ) was administered at baseline and at the last visit (Year 4; visit 9). From these FFQs, absolute values of nutrient intakes (including supplements) were calculated by NutritionQuest, utilizing primarily USDA data [23]. The DII has been previously validated with various inflammatory markers and calculated within over 40 populations, including post-menopausal women; complete descriptions of the DII are available elsewhere [11]. In short, the DII includes up to 45 parameters with individual inflammatory effect scores. A total of 29 parameters (all were nutrients) were available from the FFQs for score calculation (Table 1). The calculation for the DII is based on dietary data from a world database. This world database provides an estimate of mean and standard deviations (SD) for each inflammatory food parameter. Dietary data collected from study participants is used to calculate z-scores and centered proportions based on the world average intake, to minimize “right skewing” [11]. This proportion is centered on zero by multiplying by 2 and subtracting 1. The resulting value is multiplied by the respective food parameter effect score to create a food parameter-specific DII score. The centered proportion scores are multiplied by the corresponding food parameter effect score, creating a food parameter-specific DII score, which are summed for the overall DII score for each participant. Possible DII scores range from –8.87 to 7.98, with a more pro-inflammatory diet indicated by a higher (more positive) DII score [11]. In this study, energy-adjusted DII (E-DII™) scores also were calculated for each participant using the available assessed nutrients from the FFQs. E-DII scores are calculated per 1000 calories of food consumed, utilizing the energy-standardized version of the world database [24]. A higher, more positive, E-DII score designates a more pro-inflammatory diet whereas smaller, more negative E-DII values indicate more anti-inflammatory diets. In this analysis, the E-DII scores were the primary exposures and were analyzed as a continuous variable. E-DII scores were evaluated at both baseline and visit 9 (last visit) E-DII scores. The change in E-DII scores was calculated (Visit 9 score–Baseline score). 

*Cancer development*: The primary outcome measured in this study was first diagnosis of cancer (all types) during the study time frame, creating a dichotomous outcome (cancer yes/no). Participants were asked at each visit for any new cancer diagnoses, which were then verified by medical records, ICD-9 cancer codes, and pathology reports. New cancer status in participants was only counted once (i.e., no second primaries).

*Other measures*: Weights and heights were measured at each visit during the study and were used to calculate body mass index [BMI = weight (kg)/height (m)^2^]. BMI was categorized as <25, 25–29.9, and ≥30.0 kg/m^2^ for analysis. Other demographic information, such as age and race (White/non-White) were collected at baseline. Hormone replacement therapies (HRT) (yes/no), smoking status (never/ever), and physical activity (</≥ 150 min of moderate activity equivalents per week) were chosen as additional covariates to analyze from the RCT, for their known impact on cancer development. Physical activity data were evaluated as a combined activity-equivalent variable of moderate and vigorous reported physical activity, based off the Physical Activity Guidelines for Americans recommendation of meeting 150 min a week of moderate-intensity activity, 75 min a week of vigorous-intensity activity, or an equivalent combination of moderate- and vigorous-intensity aerobic activity [25]. HRT status was assessed from baseline medication reports, grouping together estrogen, estrogen agonist, and estrogen antagonist therapies (yes/no). Use of therapy with estrogen agonists and antagonists (such as tamoxifen or raloxifene) during the study, were primarily for treatment or prevention of osteoporosis and not for prevention of breast cancer [22]. 

*Statistical analysis*: Descriptive statistics were reported for all continuous and categorical variables (means, standard deviations, counts and percentages). E-DII scores were used for analysis to control for calorie intake. A linear mixed model (LMM) analysis on E-DII measurements with fixed effects for cancer status and time and a random effect for subjects was conducted and adjusted for multiple comparisons. Univariate and multivariate logistic regression were conducted, controlling for pertinent confounders (age, smoking status, BMI, physical activity, HRT). Race was not accounted for as a covariate due to limited group size of non-White participants (*n* = 9). Baseline E-DII scores and the E-DII change scores were included in the multivariate logistic regression to investigate the change in E-DII measurements, accounting for the baseline measurements. A power analysis was conducted for the parent study to detect reduction of cancer incidence between the intervention and control groups. They determined recruiting a sample size of 2300 and ending the study with a minimum of 1000 participants per group would be at least 80% powered, accounting for attrition [22]. No additional power analyses were performed for the present secondary analysis; analysis was performed on all available and eligible study participants. All statistical analyses were done using SAS, Version 9.4. Significance level was set at *p* < 0.05, two tailed.

## 3. Results

### 3.1. Characteristics of the Study Population

**Figure 1:**
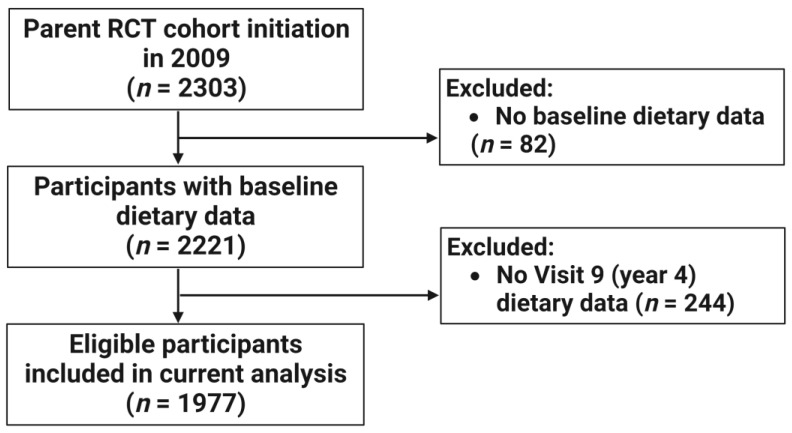
Flow diagram of participant inclusion.

Out of the total cohort of 2303 post-menopausal women, there were 2221 participants with baseline dietary data adequate to compute E-DII scores. Among them, 1977 participant records were linked to visit 9 E-DII follow-up data (see Figure 1). Therefore, this study included 1977 participants with baseline and visit 9 E-DII scores, including 1886 participants without cancer and 91 (4.6%) who developed cancer. The majority of the population was White (99.5%), with a mean age of 65 years old and mean BMI of 29.9 kg/m^2^ (Table 2). About two-thirds (67.7%) of the population had never smoked, the majority were not taking hormone replacement therapy (HRT) (81.6%), and 41.6% of participants met a physical activity level of ≥ 150 minutes of moderate activity equivalents per week. Of note, those who developed cancer were older than those who did not develop cancer (68.1 ± 7.8 vs. 65.1 ± 6.8, respectively). 

### 3.2. E-DII Scores and Cancer Development

**Figure 2:**
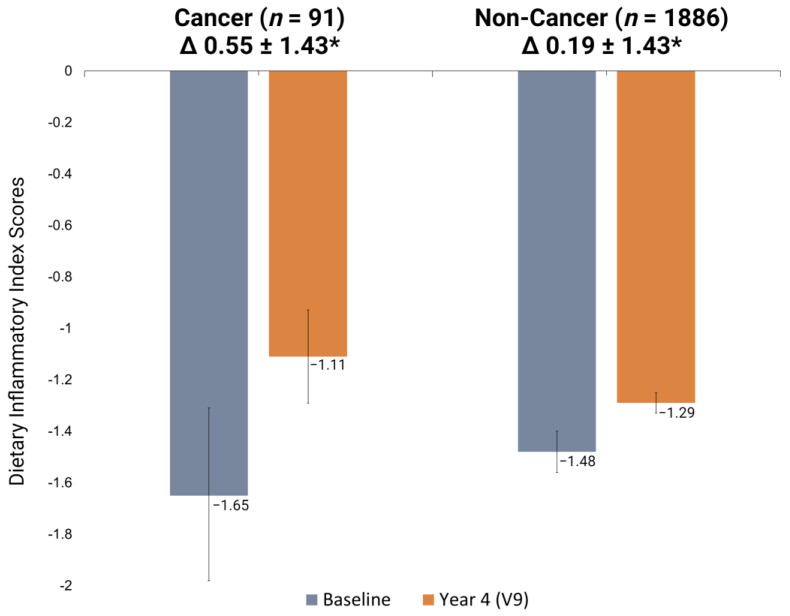
Change in mean E-DII scores between baseline and visit 9 by cancer status. * LMM for difference in mean E-DII change scores (V9–baseline) between groups; *p* = 0.02. A more negative E-DII score is more anti-inflammatory. Abbreviations: E-DII: Energy-adjusted Dietary Inflammatory Index; V9: Visit 9 (last visit in Year 4); LMM: Linear Mixed Model.

The mean baseline E-DII score for the entire cohort was −1.49 ± 1.74 and the mean visit 9 E-DII score was −1.29 ± 1.72. The difference in mean E-DII scores were tested at baseline and visit 9, as well as the difference in mean E-DII change scores (visit 9−baseline mean), between the cancer and non-cancer groups (Table 3). Mean E-DII scores were not significantly different between cancer groups at baseline (Non-cancer: −1.48 ± 1.74 vs. Cancer: −1.65 ± 1.62, *p* = 0.78) or visit 9 (Non-cancer: −1.29 ± 1.72 vs. Cancer: −1.11 ± 1.70, *p* = 0.75). There was a significant difference in the change in E-DII over time between cancer groups, with those who developed cancer having a larger, i.e., pro-inflammatory change (Figure 2; Non-cancer: Δ 0.19 ± 1.43 vs Cancer: Δ 0.55 ± 1.43; *p* = 0.02).

Logistic regression was used to further explore the association between the change in E-DII scores and cancer development (Table 4). There was a significant association between change in E-DII scores and cancer status, after controlling for the selected covariates. Specifically, the odds of cancer development in those with a larger change (more pro-inflammatory) in E-DII scores was 1.21 times the odds of the group with a smaller change (more anti-inflammatory) in E-DII scores (OR = 1.21, 95% CI = [1.02, 1.42], *p*= 0.02). Age was significantly related to cancer development, with those in the oldest age group (≥75 years) having the highest increased risk (OR = 2.45, 95% CI = [1.22, 4.92]; *p* = 0.02; data not shown). There were no significant associations between cancer groups and baseline E-DII scores, after adjustment (OR = 1.04, 95% CI = [0.90, 1.19]; *p* = 0.64). 

## 4. Discussion

Identification and treatment of specific needs of those with cancer, including dietary interventions, is a major challenge for health care professionals facing a growing cancer survivor population coinciding with the epidemic of a typical pro-inflammatory Western dietary pattern. Our findings show in a population of rural, post-menopausal women, there was a significantly larger change in E-DII scores in the participants who developed cancer, shifting to higher (more pro-inflammatory) scores after four years. Additionally, those with a higher, more pro-inflammatory change in E-DII scores had higher odds of developing cancer. While we found an increase in inflammatory potential in the diets of cancer patients over time, there was no significant association between E-DII scores and cancer status at either baseline or visit 9 time points.

Changes in DII or E-DII scores over time have not been extensively examined and those studies that have been conducted produced equivocal results. Two studies have been conducted utilizing the Women’s Health Initiative (WHI) cohort of post-menopausal women, looking at change patterns in DII scores over three years. The first study evaluated the risk of invasive breast cancer and the other, the risk of colorectal cancer. When examining breast cancer risk, there was no significant association between a DII pattern of pro-inflammatory change and risk of invasive breast cancer, after multivariate adjustment [26]. When examining colorectal cancer risk in post-menopausal women, there was a significantly higher risk of proximal colon cancer in those with a pro-inflammatory change in DII score over three years, but not in overall colorectal cancer risk [27]. A Swedish study by Bodén et al. assessed DII change over ten years and cancer risk in over 35,000 men and women with follow-up data [28]. This study found a 10-year change in DII scores was not associated with cancer risk, even in women who changed from an anti-inflammatory pattern to more pro-inflammatory pattern [28]. While the size of the total cohort was relatively large, looking at individual diet patterns severely limited the number of participants per group, with only 132 women changing from an anti-inflammatory pattern to a pro-inflammatory pattern. This may have limited the ability to observe associations as significant. These studies highlight the uncertainty of the association between change in DII scores and the association with cancer.

While the use of the DII/E-DII to assess diet changes in relation to cancer diagnosis is novel, the idea that people change their diet with a cancer diagnosis is more researched. Previous literature demonstrates a wide range in the prevalence of diet changes, which may differ by population type. Those with evident diet changes after being diagnosed with cancer ranges from 28–60%, depending on cancer type and sex [29,30,31,32,33,34]. Within the WHI cohort, 28% of post-menopausal women made diet changes after a breast cancer diagnosis [34]. Of these women, those who experienced a decrease in diet quality, assessed by the Healthy Eating Index-2010 (HEI-2010), had a significantly higher risk of death from breast cancer, after adjustment [34]. A higher HEI-2010 score would be indicative of higher intakes of fruits, vegetables, seafood and plant-proteins, and whole grains, while also minimizing intake of refined grains, saturated fat, added sugars, and sodium, which aligns with a more anti-inflammatory diet pattern. Indeed, several dietary indices, including the HEI-2010, are inversely associated with DII scores, in that as DII scores become more anti-inflammatory with lower scores, HEI-2010 scores increase, showing healthier diet patterns [35]. While over 70% of WHI cohort did not change their diet after a cancer diagnosis, it does indicate that those whose changes reflect unhealthy or pro-inflammatory eating patterns may also experience poorer health outcomes. Although the present study was unable to account for time of cancer diagnosis within the four-year timeframe, evidence from the WHI cohort suggests it may be plausible that the observed shifts to more proinflammatory diets affected the development of cancer. Conversely, it is still possible that a cancer diagnosis may lead to diet pattern changes related to treatment side effects and disease progression. Therefore, it would be important to establish a timeline of events, with yearly diet assessments, in future studies to distinguish a causal relationship.

In contrast to our study, DII scores with higher inflammatory potential have been associated with several types of cancers, including but not limited to colorectal cancer [36,37], prostate cancer [38], renal cancer [38], ovarian cancer [15,39], and breast cancer [13,38,40]. A recent meta-analysis reviewing 21 studies of DII and breast cancer showed while there was an overall 16% increased risk of breast cancer in those with the most pro-inflammatory DII scores, the six studies that used E-DII scores revealed no association with breast cancer risk [41]. Authors noted that the use of E-DII as the independent variable has been utilized infrequently, which could lead to this finding [41]. As energy intake is strongly associated with DII scores, the E-DII score was created to control for energy intake at the source of the data that is used to create the DII scores [24]. Outside of breast cancer, the E-DII score has been associated with increased risk of other cancers [42] and other disease states [43]. 

Several mechanisms for how diet impacts cancer development have been explored, including the interdependent relationship between inflammatory markers and oxidative stress [44,45]. Oxidative stress is broadly defined as the imbalance of oxidants and antioxidants, where the level of free radicals, including reactive oxygen species (ROS) is not overcome by the antioxidant system. The inflammatory process encompasses an array of physiologic responses, ultimately to resolve cell injury in the acute setting. However, chronic inflammation has been associated with damage to DNA and tissues, promoting cancer development [3]. Conditions driven by oxidative stress in turn influence systemic inflammation through enhancing pro-inflammatory gene expression, including the production of the pro-inflammatory cytokine TNF-α [44,45]. Conversely, conditions instigated by an inflammatory process also contributes to oxidative stress through the creation of ROS and antioxidant depletion [45]. This interconnected relationship between inflammation and oxidative stress is important to understand in the context of the role of diet. 

For example, diets high in fiber, a DII component, have been associated with cancer prevention and reduced cancer mortality [46,47,48]. Through colonic microbial fermentation of fiber, short-chain fatty acids (SCFA) are produced [49]. SCFA in turn can influence systemic inflammation through activation of G-protein receptors and promotion of histone acetylation [44], as well as impact oxidative stress through the regulation of oxidoreductase and restoration of the antioxidant, glutathione [50,51]. Through these mechanisms, SCFA have been associated with inhibitory effects on colon cancer [52]. The DII components also include several known antioxidants including vitamins A, C, E, beta-carotene, zinc, and several polyphenols. High-antioxidant dietary patterns, like plant-based diets, are often sought as a cancer prevention intervention to impact inflammation and oxidative stress. The Meat and Three Greens (M3G) Feasibility Trial examined the impact of daily leafy green consumption in those at risk of colorectal cancer on oxidative DNA damage and inflammation. After 4 weeks of consuming leafy green vegetables, both markers of oxidative DNA damage (plasma and fecal 8OHdG) and inflammation (TNF-α) significantly decreased, compared to controls [53]. A recent RCT by Gualtieri et al., examined the impact of supplementing healthy subjects with an antioxidant-rich, food-derived juice drink while following a Mediterranean diet, compared to the control of just the Mediterranean diet over two weeks. Those with the addition of the antioxidant-rich juice positively improved the oxidative and inflammatory gene expression, including *Superoxide dismutase* (*SOD1*) and *Peroxisome Proliferator-Activated Receptor γ* (*PPARγ*) [54]. These studies illustrate the biological plausibility of using realistic dietary interventions to impact inflammation and oxidative stress mechanisms in the short term. Further investigation should be conducted to determine the ultimate impact on health status and disease prevention over time. While individual dietary components may contribute to cancer prevention, the DII integrates the synergistic effect of multiple nutrients and foods together that have been associated with inflammatory markers. The DII has additional preliminary associations with markers of oxidative stress including serum malondialdehyde and total antioxidant capacity [55]. However, a greater understanding of the mechanisms associated with diet changes indicating high inflammatory potential can impact cancer development is needed.

Our study did have a sufficiently large cohort and the original RCT was well powered to see cancer incidence but was comparatively smaller than other post-menopausal women cohorts examining DII and cancer. For example, a study utilizing the Iowa Women’s Health Study included 34,700 women and 2910 breast cancer cases [56]. Our present study was only able to examine 91 cancer cases among 1977 women, which may have affected our ability to see an association. A rural population may also introduce additional factors that were not accounted for in this study, including education status and other social determinants of health, which have been associated with higher DII scores further limiting our ability to see an association [13,27]. Further research should be conducted to compare changes in DII scores in rural versus urban populations, as they may face distinct challenges in improving health and preventing chronic disease. Additionally, other studies focusing on DII in post-menopausal women have shown higher mean inflammatory potential, compared to our mean E-DII scores [13,27,56]. This suggests our study population had more anti-inflammatory diets overall, which could have prevented us from seeing an association between cancer development and E-DII scores.

This study is strengthened by having baseline and follow up data but limited by its small number of cancer diagnoses and diversity, with almost all participants being White, affecting generalizability to other populations. Timepoints of when cancer was diagnosed were not available for this analysis. This limits our understanding of when cancer was diagnosed in relation to the shift in dietary changes and should be assessed in future studies. Recall bias could also affect the reports in the FFQs, which are known to be associated with response set biases. Among these, social desirability seems to be more strongly expressed in women than in men, skewing nutrient intakes from which E-DII scores were calculated [57,58,59]. Our study may be a biased representation of cancer cases compared to the original study, as our study does not include those who developed cancer but did not have follow-up data. 

## 5. Conclusions

Analyzing the change in E-DII scores in association with cancer risk has seldom been evaluated. Our pilot data shows that a significant change in an individual’s diet toward a more pro-inflammatory pattern increases the odds of a diagnosis of cancer. However, how pro-inflammatory shifts in E-DII scores affects health outcomes during cancer survivorship is relatively unknown. Therefore, this research can serve as a foundation for interventional trials in cancer survivorship to determine if implementing anti-inflammatory diet patterns can improve health outcomes and contribute to new body of evidence for health care professionals, transforming diet and lifestyle education for cancer survivors. 

## Figures and Tables

**Table 1:** DII components available for calculation of the DII score * [9].

Alcohol	Energy (kcal)	MUFA	Riboflavin	Vitamin C
Vitamin B12	Total Fat	Niacin	Saturated Fat	Vitamin D
Vitamin B6	Fiber	n-3 fatty acids	Selenium	Vitamin E
β- Carotene	Folic Acid	n-6 fatty acids	Thiamin	Zinc
Carbohydrate	Iron	Protein	Trans fat	Isoflavones
Cholesterol	Magnesium	PUFA	Vitamin A	
* 29 of 45 components available for calculation. Components not available for calculation: caffeine, eugenol, garlic, ginger, onion, saffron, turmeric, green/black tea, flavon-3-ol, flavones, flavonols, flavonones, anthocyanidins, pepper, thyme/oregano, rosemary.				

**Table 2:** Baseline Characteristics of Total Study Population and by Cancer Status.

Characteristics	Total Population (*n* = 1977)	Non-Cancer (*n* = 1886)	Cancer (*n* = 91)
Age (years; Mean ± Std.)	65.2 ± 6.8	65.1 ± 6.8	68.1 ± 7.8
Age (years) 55–59 60–64 65–74 ≥75	529 (26.8) 579 (29.3) 656 (33.2) 213 (10.8)	511 (27.1) 565 (30.0) 615 (32.6) 195 (10.3)	18 (19.8) 14 (15.4) 41 (45.0) 18 (19.8)
Race White Non-white/not available	9 (0.5) 1968 (99.5)	9 (0.5) 1877 (99.5)	0 91 (100)
BMI (kg/m^2^; Mean ± Std.)	29.9 ± 6.5	29.9 ± 6.5	29.0 ± 6.5
BMI (kg/m^2^) <25.0 25–29.9 ≥30.0	465 (23.6) 667 (33.8) 842 (42.6)	439 (23.3) 634 (33.7) 810 (43.0)	26 (28.6) 33 (36.3) 32 (35.2)
Smoking Never Ever	1338 (67.7) 639 (32.3)	1278 (67.8) 608 (32.2)	60 (65.9) 31 (34.1)
Physical activity (min) <150 ≥150	1146 (58.4) 815 (41.6)	650 (34.7) 1223 (65.3)	23 (26.1) 65 (73.9)
HRT No Yes	1614 (81.6) 363 (18.4)	1537 (81.5) 349 (18.5)	77 (84.6) 14 (15.4)

Abbreviations: BMI: Body Mass Index; HRT: Hormone replacement therapy.

**Table 3:** Difference in E-DII scores between cancer groups.

E-DII (Mean ± Std.)	Non-Cancer (*n* = 1886)	Cancer (*n* = 91)	*p*-Value *
**Baseline**	–1.48 ± 1.74	–1.65 ± 1.62	0.78
**Visit 9**	–1.29 ± 1.72	–1.11 ± 1.70	0.75
**E-DII change (V9–baseline)**	0.19 ± 1.43	0.55 ± 1.43	0.02

* raw mean+ Std. values are displayed; *p*-values derived from LMM. Abbreviations: E-DII: Energy-adjusted Dietary Inflammatory Index; V9: Visit 9 (last visit in Year 4); LMM: Linear Mixed Model; Std: standard deviation.

**Table 4:** Unadjusted and multiple logistic regression of cancer development (*n* = 1977).

E-DII	Unadjusted OR [95% CI]	*p*-Value	Adjusted OR * [95% CI]	*p*-Value
E-DII Baseline E-DII V9 visit E-DII change (V9–baseline)	0.94 [0.83, 1.06] 1.07 [0.94 1.20] 1.20 [1.04 1.40]	0.31 0.31 0.01	1.04 [0.90, 1.19] - 1.21 [1.02, 1.42]	0.64 - 0.02

Abbreviations: E-DII: Energy-adjusted Dietary Inflammatory Index; V1: Baseline visit; V9: Visit 9 (last visit). * Multivariate logistic regression model adjusted for: age, smoking status, BMI, physical activity, hormone replacement therapy.

## Data Availability

Not applicable.

## References

[1] Deng T., Lyon C.J., Bergin S., Caligiuri M.A., Hsueh W.A. (2016). Obesity, Inflammation, and Cancer. Annu. Rev. Pathol..

[2] Murata M. (2018). Inflammation and cancer. Environ. Health Prev. Med..

[3] World Cancer Research Fund/American Institute for Cancer Research Continuous Update Project Expert Report 2018: The Cancer Process. www.dietandcancerreport.org.

[4] Aleksandrova K., Koelman L., Rodrigues C.E. (2021). Dietary patterns and biomarkers of oxidative stress and inflammation: A systematic review of observational and intervention studies. Redox Biol..

[5] Chrysohoou C., Panagiotakos D.B., Pitsavos C., Das U.N., Stefanadis C. (2004). Adherence to the Mediterranean diet attenuates inflammation and coagulation process in healthy adults: The ATTICA Study. J. Am. Coll. Cardiol..

[6] Estruch R., Martinez-Gonzalez M.A., Corella D., Salas-Salvado J., Ruiz-Gutierrez V., Covas M.I., Fiol M., Gomez-Gracia E., Lopez-Sabater M.C., Vinyoles E. (2006). Effects of a Mediterranean-style diet on cardiovascular risk factors: A randomized trial. Ann. Intern. Med..

[7] Turpin W., Dong M., Sasson G., Raygoza Garay J.A., Espin-Garcia O., Lee S.H., Neustaeter A., Smith M.I., Leibovitzh H., Guttman D.S. (2022). Mediterranean-Like Dietary Pattern Associations With Gut Microbiome Composition and Subclinical Gastrointestinal Inflammation. Gastroenterology.

[8] Barbaresko J., Koch M., Schulze M.B., Nöthlings U. (2013). Dietary pattern analysis and biomarkers of low-grade inflammation: A systematic literature review. Nutr. Rev..

[9] Johansson-Persson A., Ulmius M., Cloetens L., Karhu T., Herzig K.H., Onning G. (2014). A high intake of dietary fiber influences C-reactive protein and fibrinogen, but not glucose and lipid metabolism, in mildly hypercholesterolemic subjects. Eur. J. Nutr..

[10] Wood A.D., Strachan A.A., Thies F., Aucott L.S., Reid D.M., Hardcastle A.C., Mavroeidi A., Simpson W.G., Duthie G.G., Macdonald H.M. (2014). Patterns of dietary intake and serum carotenoid and tocopherol status are associated with biomarkers of chronic low-grade systemic inflammation and cardiovascular risk. Br. J. Nutr..

[11] Shivappa N., Steck S.E., Hurley T.G., Hussey J.R., Hebert J.R. (2014). Designing and developing a literature-derived, population-based dietary inflammatory index. Public Health Nutr..

[12] Hayati Z., Montazeri V., Shivappa N., Hebert J.R., Pirouzpanah S. (2022). The association between the inflammatory potential of diet and the risk of histopathological and molecular subtypes of breast cancer in northwestern Iran: Results from the Breast Cancer Risk and Lifestyle study. Cancer.

[13] Shivappa N., Blair C.K., Prizment A.E., Jacobs D.R., Hebert J.R. (2017). Prospective study of the dietary inflammatory index and risk of breast cancer in postmenopausal women. Mol. Nutr. Food Res..

[14] Shivappa N., Hébert J.R., Rosato V., Montella M., Serraino D., La Vecchia C. (2017). Association between the dietary inflammatory index and breast cancer in a large Italian case-control study. Mol. Nutr Food Res..

[15] Shivappa N., Hebert J.R., Rosato V., Rossi M., Montella M., Serraino D., La Vecchia C. (2016). Dietary inflammatory index and ovarian cancer risk in a large Italian case-control study. Cancer Causes Control.

[16] Shivappa N., Sandin S., Löf M., Hébert J.R., Adami H.O., Weiderpass E. (2015). Prospective study of dietary inflammatory index and risk of breast cancer in Swedish women. Br. J. Cancer.

[17] Tabung F.K., Steck S.E., Zhang J., Ma Y., Liese A.D., Agalliu I., Hingle M., Hou L., Hurley T.G., Jiao L. (2015). Construct validation of the dietary inflammatory index among postmenopausal women. Ann. Epidemiol..

[18] Siegel R.L., Miller K.D., Wagle N.S., Jemal A. (2023). Cancer statistics, 2023. CA Cancer J. Clin..

[19] Miller P.E., Morey M.C., Hartman T.J., Snyder D.C., Sloane R., Cohen H.J., Demark-Wahnefried W. (2012). Dietary patterns differ between urban and rural older, long-term survivors of breast, prostate, and colorectal cancer and are associated with body mass index. J. Acad. Nutr. Diet..

[20] Trivedi T., Liu J., Probst J., Merchant A., Jhones S., Martin A.B. (2015). Obesity and obesity-related behaviors among rural and urban adults in the USA. Rural Remote Health.

[21] Bhatia S., Landier W., Paskett E.D., Peters K.B., Merrill J.K., Phillips J., Osarogiagbon R.U. (2022). Rural-Urban Disparities in Cancer Outcomes: Opportunities for Future Research. J. Natl. Cancer Inst..

[22] Lappe J., Watson P., Travers-Gustafson D., Recker R., Garland C., Gorham E., Baggerly K., McDonnell S.L. (2017). Effect of Vitamin D and Calcium Supplementation on Cancer Incidence in Older Women: A Randomized Clinical Trial. JAMA.

[23] Bowman S.A., Friday J.E., Moshfegh A.J. (2008). MyPyramid Eqivalents Database, 2.0 for USDA Survey Foods 2003–2004.

[24] Hebert J.R., Shivappa N., Wirth M.D., Hussey J.R., Hurley T.G. (2019). Perspective: The Dietary Inflammatory Index (DII)-Lessons Learned, Improvements Made, and Future Directions. Adv. Nutr..

[25] USDA Department of Health and Human Services, DHHS (2018). Physical Activity Guidelines for Americans.

[26] Tabung F.K., Steck S.E., Liese A.D., Zhang J., Ma Y., Johnson K.C., Lane D.S., Qi L., Snetselaar L., Vitolins M.Z. (2016). Patterns of change over time and history of the inflammatory potential of diet and risk of breast cancer among postmenopausal women. Breast Cancer Res. Treat..

[27] Tabung F.K., Steck S.E., Ma Y., Liese A.D., Zhang J., Lane D.S., Ho G.Y.F., Hou L., Snetselaar L., Ockene J.K. (2017). Changes in the Inflammatory Potential of Diet Over Time and Risk of Colorectal Cancer in Postmenopausal Women. Am. J. Epidemiol..

[28] Boden S., Myte R., Wennberg M., Harlid S., Johansson I., Shivappa N., Hebert J.R., Van Guelpen B., Nilsson L.M. (2019). The inflammatory potential of diet in determining cancer risk; A prospective investigation of two dietary pattern scores. PLoS ONE.

[29] Bours M.J., Beijer S., Winkels R.M., van Duijnhoven F.J., Mols F., Breedveld-Peters J.J., Kampman E., Weijenberg M.P., van de Poll-Franse L.V. (2015). Dietary changes and dietary supplement use, and underlying motives for these habits reported by colorectal cancer survivors of the Patient Reported Outcomes Following Initial Treatment and Long-Term Evaluation of Survivorship (PROFILES) registry. Br. J. Nutr..

[30] Ghelfi F., Tieri M., Gori S., Nicolis F., Petrella M.C., Filiberti A., Apolone G., Titta L. (2018). Do cancer patients change their diet in the e-health information era? A review of the literature and a survey as a proposal for the Italian population. Food Res. Int..

[31] Hagen K.B., Aas T., Kvaloy J.T., Soiland H., Lind R. (2018). Diet in women with breast cancer compared to healthy controls—What is the difference?. Eur. J. Oncol. Nurs..

[32] Maunsell E., Drolet M., Brisson J., Robert J., Deschenes L. (2002). Dietary change after breast cancer: Extent, predictors, and relation with psychological distress. J. Clin. Oncol..

[33] Patterson R.E., Neuhouser M.L., Hedderson M.M., Schwartz S.M., Standish L.J., Bowen D.J. (2003). Changes in diet, physical activity, and supplement use among adults diagnosed with cancer. J. Am. Diet. Assoc..

[34] Sun Y., Bao W., Liu B., Caan B.J., Lane D.S., Millen A.E., Simon M.S., Thomson C.A., Tinker L.F., Van Horn L.V. (2018). Changes in Overall Diet Quality in Relation to Survival in Postmenopausal Women with Breast Cancer: Results from the Women’s Health Initiative. J. Acad. Nutr. Diet..

[35] Wirth M.D., Hebert J.R., Shivappa N., Hand G.A., Hurley T.G., Drenowatz C., McMahon D., Shook R.P., Blair S.N. (2016). Anti-inflammatory Dietary Inflammatory Index scores are associated with healthier scores on other dietary indices. Nutr. Res..

[36] Fan Y., Jin X., Man C., Gao Z., Wang X. (2017). Meta-analysis of the association between the inflammatory potential of diet and colorectal cancer risk. Oncotarget.

[37] Tabung F.K., Steck S.E., Ma Y., Liese A.D., Zhang J., Caan B., Hou L., Johnson K.C., Mossavar-Rahmani Y., Shivappa N. (2015). The association between dietary inflammatory index and risk of colorectal cancer among postmenopausal women: Results from the Women’s Health Initiative. Cancer Causes Control.

[38] Moradi S., Issah A., Mohammadi H., Mirzaei K. (2018). Associations between dietary inflammatory index and incidence of breast and prostate cancer: A systematic review and meta-analysis. Nutrition.

[39] Nagle C.M., Ibiebele T., Shivappa N., Hebert J.R., DeFazio A., Webb P.M., Australian Ovarian Cancer Study (2019). The association between the inflammatory potential of diet and risk of developing, and survival following, a diagnosis of ovarian cancer. Eur. J. Nutr..

[40] Wang L., Liu C., Zhou C., Zhuang J., Tang S., Yu J., Tian J., Feng F., Liu L., Zhang T. (2019). Meta-analysis of the association between the dietary inflammatory index (DII) and breast cancer risk. Eur. J. Clin. Nutr..

[41] Hayati Z., Jafarabadi M.A., Pirouzpanah S. (2022). Dietary inflammatory index and breast cancer risk: An updated meta-analysis of observational studies. Eur. J. Clin. Nutr..

[42] Mazul A.L., Shivappa N., Hébert J.R., Steck S.E., Rodriguez-Ormaza N., Weissler M., Olshan A.F., Zevallos J.P. (2018). Proinflammatory diet is associated with increased risk of squamous cell head and neck cancer. Int. J. Cancer.

[43] Mazidi M., Shivappa N., Wirth M.D., Hebert J.R., Kengne A.P. (2018). Greater Dietary Inflammatory Index score is associated with higher likelihood of chronic kidney disease. Br. J. Nutr..

[44] Liu P., Wang Y., Yang G., Zhang Q., Meng L., Xin Y., Jiang X. (2021). The role of short-chain fatty acids in intestinal barrier function, inflammation, oxidative stress, and colonic carcinogenesis. Pharmacol. Res..

[45] Biswas S.K. (2016). Does the Interdependence between Oxidative Stress and Inflammation Explain the Antioxidant Paradox?. Oxid. Med. Cell. Longev..

[46] Aune D., Chan D.S., Lau R., Vieira R., Greenwood D.C., Kampman E., Norat T. (2011). Dietary fibre, whole grains, and risk of colorectal cancer: Systematic review and dose-response meta-analysis of prospective studies. BMJ.

[47] Gaesser G.A. (2020). Whole Grains, Refined Grains, and Cancer Risk: A Systematic Review of Meta-Analyses of Observational Studies. Nutrients.

[48] Molina-Montes E., Ubago-Guisado E., Petrova D., Amiano P., Chirlaque M.D., Agudo A., Sánchez M.J. (2021). The Role of Diet, Alcohol, BMI, and Physical Activity in Cancer Mortality: Summary Findings of the EPIC Study. Nutrients.

[49] Hamer H.M., Jonkers D.M., Bast A., Vanhoutvin S.A., Fischer M.A., Kodde A., Troost F.J., Venema K., Brummer R.J. (2009). Butyrate modulates oxidative stress in the colonic mucosa of healthy humans. Clin. Nutr..

[50] Ebert M.N., Klinder A., Peters W.H., Schäferhenrich A., Sendt W., Scheele J., Pool-Zobel B.L. (2003). Expression of glutathione S-transferases (GSTs) in human colon cells and inducibility of GSTM2 by butyrate. Carcinogenesis.

[51] Knapp B.K., Bauer L.L., Swanson K.S., Tappenden K.A., Fahey G.C., De Godoy M.R. (2013). Soluble fiber dextrin and soluble corn fiber supplementation modify indices of health in cecum and colon of Sprague-Dawley rats. Nutrients.

[52] Tang Y., Chen Y., Jiang H., Robbins G.T., Nie D. (2011). G-protein-coupled receptor for short-chain fatty acids suppresses colon cancer. Int. J. Cancer.

[53] Frugé A.D., Smith K.S., Riviere A.J., Tenpenny-Chigas R., Demark-Wahnefried W., Arthur A.E., Murrah W.M., van der Pol W.J., Jasper S.L., Morrow C.D. (2021). A Dietary Intervention High in Green Leafy Vegetables Reduces Oxidative DNA Damage in Adults at Increased Risk of Colorectal Cancer: Biological Outcomes of the Randomized Controlled Meat and Three Greens (M3G) Feasibility Trial. Nutrients.

[54] Gualtieri P., Marchetti M., Frank G., Smeriglio A., Trombetta D., Colica C., Cianci R., De Lorenzo A., Di Renzo L. (2023). Antioxidant-Enriched Diet on Oxidative Stress and Inflammation Gene Expression: A Randomized Controlled Trial. Genes.

[55] Moradi F., Heidari Z., Teimori A., Ghazvini M., Imani Z.F., Naeini A.A. (2022). The Association Between the Dietary Inflammatory Index (DII) and Some Serum Oxidative Stress Markers in Non-Alcoholic Fatty Liver Disease: Case-Control. Int. J. Prev. Med..

[56] Shivappa N., Blair C.K., Prizment A.E., Jacobs D.R., Hebert J.R. (2018). Dietary inflammatory index and risk of renal cancer in the Iowa Women’s Health Study. Eur. J. Nutr..

[57] Hebert J.R. (2016). Social Desirability Trait: Biaser or Driver of Self-Reported Dietary Intake?. J. Acad. Nutr. Diet..

[58] Hebert J.R., Ebbeling C.B., Matthews C.E., Hurley T.G., Ma Y., Druker S., Clemow L. (2002). Systematic errors in middle-aged women’s estimates of energy intake: Comparing three self-report measures to total energy expenditure from doubly labeled water. Ann. Epidemiol..

[59] Hebert J.R., Ma Y., Clemow L., Ockene I.S., Saperia G., Stanek E.J., Merriam P.A., Ockene J.K. (1997). Gender differences in social desirability and social approval bias in dietary self-report. Am. J. Epidemiol..

